# Experiencing objectified health: turning the body into an object of attention

**DOI:** 10.1007/s11019-020-09949-0

**Published:** 2020-04-03

**Authors:** Bas de Boer

**Affiliations:** Philosophy Department, University of Twente, Enschede, The Netherlands

**Keywords:** Phenomenology of health, Postphenomenology, Technological mediation, Self-tracking, Digital twin

## Abstract

In current phenomenology of medicine, health is often understood as a state of transparency in which our body refrains from being an object of explicit attention. In this paper, I argue that such an understanding of health unnecessarily presupposes an overly harmonious alignment between subjective and objective body, resulting in the idea that our health remains phenomenologically inaccessible. Alternatively, I suggest that there are many occasions in which one’s body in health does become an object of attention, and that technologies mediate how a relation with one’s body is formed. First, I show prominent accounts in current phenomenology of medicine understand health in terms of a harmonious alignment between objective and subjective body. Second, I argue that there are many occasions in which there is a disharmony between objective and subjective body, and suggest that also in health, we cannot escape being an object that we often relate to. Then, I draw on postphenomenology to show how technologies such as digital self-tracking applications and digital twins can be understood as mediating the relationship with one’s own body in a specific way. In conclusion, I argue that both technologies make present the objective body as a site for hermeneutic inquiry such that it can be interacted with in terms of health parameters. Furthermore, I point to some relevant differences in how different technologies make aspects of our own body phenomenologically present.

## Introduction

In current phenomenology of medicine, health is often understood as a state of transparency in which our body refrains from being an object of explicit attention. This view has been famously summarized in the remark by the French physician René Leriche that “health is life lived in the silence of the organs” (op. cit. Canguilhem 1991, p. 91). In this paper, I argue that such an understanding of health unnecessarily presupposes an overly harmonious alignment between subjective and objective body, resulting in the idea that our health remains phenomenologically inaccessible. Alternatively, I pose the question of how health becomes experientially present in the (often not harmonious) interaction between subjective and objective body.

Exploring how our health becomes experientially present to us is important because medicine has become increasingly focused on the prevention of future illnesses. For example, the use of digital self-tracking applications that allows for monitoring one’s daily number of steps or calorie intake is promoted as a means to adopt a more healthy lifestyle. This development, then, has prompted researchers to understand them as an inversion of what Hans-Georg Gadamer has called *The Enigma of Health* (1996), because self-tracking applications create “a constant awareness of our health status which is necessary to sculpt our everyday eating and living patterns in order to contain the epidemic of chronic diseases” (Moerenhout et al. 2018, p. 39). These applications thus can be said to embody a normative imperative, because they draw health out of its transparency, thereby construing it as something demanding our constant attention (Hofmann and Svenaeus 2018, p. 11). Accordingly, such technologies invite asking *how* our experience of health changes as a result of their presence.

A central distinction in the phenomenology of health is the one between *subjective body* and *objective body* that can be traced back to the appearance of the terms *Leib* (subjective body) and *Körper* (objective body) in the work of Husserl (e.g., 1989). The latter refers to our body as being an object in geometrical space that can be observed from a third-person perspective, and as having certain physiological characteristics that can be measured, such that it can become an object of biomedical investigation. The former captures how we experience the world *through* our body, and how it is constitutive of how we understand ourselves and the world around us. We relate to the world *through* our subjective body, such that it “is the vehicle of being in the world […] [through which we are] united with a definite milieu, merg[e] with certain projects, and [are] perpetually engaged therein” (Merleau-Ponty 2012, p. 84). The subjective body is what links us to the world and evades biomedical objectification, and can only be accessed from a first-person perspective. In everyday healthy experience, these two are often thought to be harmoniously aligned, such that “in the smooth everyday experience of a healthy body, the body as object and the body as subject are aligned and experienced as harmonious.[…] The fundamental bodily experience of health is one of harmony, control, and predictability” (Carel 2016, p. 55).

However, there are multiple instances in which I explicitly experience my body as an object. I might simply look at it out of curiosity, or ask whether it would look more attractive when exercising more often. Furthermore, I can perceive my own organs or physiology through CT or MRI scans. And, nowadays, I can monitor my daily calorie intake or number of steps taken by using health monitoring applications. Furthermore, medicine promises the future possibility to develop my digital twin: a personalized model of myself and my biology that is being updated in light of measurements of health and lifestyle parameters, as well as of my medical history (Brown 2016; El Saddik 2018). In sum, it seems to occur frequently in our everyday experience that we (will) experience our objective body as something we are not harmoniously aligned with, yet as belonging to us (Slatman 2009, 2014). This suggests that experiencing oneself as healthy involves experiencing the disruption of the harmony of subjective and objective aspects of one’s body.

The promotion and use of digital self-tracking applications and/or digital twins explicitly invites constituting one’s own body as an object. However, there seems to be a difference between looking at my hand out of curiosity and the constitution of my own body when using self-tracking applications, or when encountering ‘my’ digital twin. But what is this difference? Philosophers of technology working in the postphenomenological tradition have focused on how technologies shape our understanding of ourselves and the world around us. Using the notion of “technological mediation,” they typically stress that technologies are no neutral intermediaries allowing to better execute pre-defined goals and projects, but actively mediate how the world becomes present to us (e.g., Ihde 1990; Verbeek 2005). In this paper, I show how this notion can be fruitfully applied for phenomenologically understanding how technologies shape the interaction between subjective body and objective body by co-constituting as what kind of project our own body becomes present.

The paper is structured as follows: First, I show how health is understood in terms of a harmonious alignment between objective and subjective body in prominent accounts in current phenomenology of medicine. Second, I argue that there are many occasions in which there is a disharmony between objective and subjective body, and suggest that also in health, we cannot escape being an object that we relate to. Then, I draw on postphenomenology to show how technologies such as digital self-tracking applications and digital twins can be understood as each mediating the relationship with one’s own objective body in a specific way. In conclusion, I argue that both technologies mediate our own body in such a way that they give rise to an experience of *objectified health*: they make present the objective body as a site for hermeneutic inquiry that can be interacted with in terms of health parameters. Furthermore, I point to some relevant differences in how different technologies make aspects of our own body phenomenologically present.

## Health as harmonious alignment

In this section, I show how in the work of Havi Carel and Fredrik Svenaeus, two prominent current phenomenologists of medicine, it is assumed that being healthy requires our objective body to become transparent in our experience (i.e., is not explicitly thematized as an object of concern).

### Health and bodily certainty

According to Carel, the experience of the healthy body is characterized by its *transparency*. For example, when eating, what I experience are neither the bodily movements required to move food from my plate into my mouth, nor the way in which my digestive system processes what I am eating. Instead, I am engaged in the activity of eating and immersed in the taste of the food without paying attention to the bodily movements and processes involved in this activity. Accordingly, although my body always is both subject and object, I do not experience it *as* an object in the ordinary flow of healthy life.

Carel draws on analogy between the transparency of our body in health and Martin Heidegger’s phenomenology of tool use. Heidegger makes a distinction between present-at-hand entities and ready-to-hand entities. According to him, we do not relate to the tools we use as object in front of us (i.e., as present-at-hand), but we rather experience the world *through* them when pursuing our projects (ready-to-hand). It is only when tools cease to work that they appear as present-at-hand entities that are explicitly thematized as objects (e.g., Heidegger 1985, §§15–16). For example, when I am writing a letter, I do not experience the pen I am writing with, but experience the surface of the piece of paper that I am writing on *through* the pen, while being immersed in the activity of writing a letter. It is only when the pen stops working properly that it is explicitly thematized as an object of attention, inviting questions such as: “why does this object prevent me from further engagement into my project?”.

Carel extends Heidegger’s analysis of the breakdown of tools to the body by inviting to “imagine that the pen works perfectly but I cannot use my hand […].” She argues that “in these cases, too, I experience a failure of a tool, but this time the tool is part of my body” (Carel 2016, p. 61). Carel’s point is of course not to place bodily disfunctioning and the breakdown of a tool on equal footing, but builds on Heidegger’s phenomenology of tool use to show that bodily disfunctioning modifies our experience of the world. The crucial difference that becomes visible when Heidegger’s phenomenology of tool use is extended to the human body is that disfunctioning body parts (often) remain attached to the human body, and tend to become increasingly disabling. Because of this, bodily disfunctioning has a profoundly different temporal dimension than the breakdown of a tool, having the potential to transform our experience and understanding of ourselves and the environment definitively. What this points to is that our experience of the world in health is dependent on the integrity of our bodies, such that disruptions of this integrity reveal the contingency of our everyday relation with the world (Ibid., p. 62). Only when our body becomes a burden do we thematize our body as influencing our relationship with the environment.

Carel argues that simple bodily disruptions such as a cold or headache do not radically transform our healthy, everyday being in the world, because these still appear within a totality of *bodily certainty*. That is, “we feel tacitly confident (or rather, we do not normally question) that our bodies will continue to function in a similar fashion in which they have in the past” (Ibid., p. 89). The habits that we have developed throughout our lives constitute a meaningful whole that is not directly experienced, yet implicitly offers a horizon against which the things we experience and the projects we engage in appear. For example, I expect that my legs will be able to walk me to the bus station this afternoon, which allows me to travel back home and play football in the evening. This I usually do not reflect upon, but is constitutive of how I understand my relation to myself and the environment. Also, when I am suffering from a headache, these activities remain to appear within a similar meaningful whole. It might take slightly longer to walk to the bus stop or be somewhat less pleasant, or even I must skip playing football today. Yet, I remain to expect that next week or the week after I can perform these activities again. Also in such cases, the world remains familiar to me because I tacitly assume that I am capable of remaining to execute such habits and expect that my body does not disrupt the routinely habitual way through which I understand myself and my environment.

Carel thus conceives of health as a state of harmony between objective and subjective body, such that the former is transparent due to a sense of bodily certainty. This harmony makes possible the effortless execution of developed habits and constitutes a bodily certainty that makes it that we take it for granted that we can remain doing so in the future. As such, our objective body *as* a healthy body refrains from becoming an object of experience. Instead, *through* it, we experience and understand ourselves and the world in an unproblematic manner, thereby orienting to a meaningful horizon that allows for the planning and carrying out of our projects.

### Health as homelikeness

In this section, I will turn to the work of Svenaeus in order to analyze if a phenomenology of the health can depart ‘from within health’. Svenaeus draws on the work of Martin Heidegger to show that our everyday engagement with the world already offers a foundation for a phenomenology of health. Just as Carel characterizes health in terms of the transparency of the body, Svenaeus maintains that health is something we transparently live *through*, instead of something that we consciously attend to. Therefore, a phenomenology of health requires an investigation of “the meaning of human experience situated in the world as acting, attuned, and embodied” (Svenaeus 2001, p. 82), which is why Svenaeus uses Heidegger’s work as a starting-point. In doing so, he holds that, on an ontological level, human beings (*Dasein*) must be understood in terms of their being-in-the-world; first and foremost, the world does not appear to a human being as an external object, but as an already meaningful totality to which (s)he is open to. Through this openness, the totality appears as a pattern for acting, thinking, feeling, and talking. Crucial for Svenaeus—and Heidegger—is that understanding a totality as meaningful is not a cognitive disposition that can be explicitly articulated, but a form of *attunement*. To understand, then, “is to find one’s place in the meaning-structure of the world and project oneself towards possible goals” (Ibid., p. 88).

Through attuned understanding, human beings realize themselves in the world as individuals projected in a particular way. However, at the same time, individuals are never fully familiar with (i.e., at home in) their world because it is shared with other individuals and a nature that somehow resists understanding. Necessarily, our familiarity with the world is always “pervaded by a homelessness” (Ibid., p. 93) that threatens to alienate us from our everyday being-at-the-world. Health, according to Svenaeus, is the keeping at bay of this alienation; it “is to be understood as a being at home that keeps the not being at home in the world from becoming apparent” (Ibid., p. 93). Accordingly, health is not an entity that can be pointed at, or a specific state of affairs that can be analyzed, but is a *process* of homelike attunement in which being-at-home is balanced with not-being-at-home such that our experience of being in the world is one of familiarity and continuity.

Because health is defined as a balancing process, Svenaeus claims that health can be phenomenologically accessed by analyzing the balancing of homelikeness and unhomelikeness. He compares it with riding a bicycle during which your balance may be challenged due to the surface you are riding on, and in reaction to which you will attempt to regain balance in order not to fall down, successfully or not (Ibid., p. 95). Similarly, our balancing can be challenged when experiencing an episode of migraine, during which the world is no longer familiar because vision is blurred and light no longer appears as a transparent source allowing us to engage in our daily activities (e.g., walking, typing), but instead as a source of frustration that increases the intensity of the migraine episode. In such moments we are attuned with the world in such a way that it no longer appears as familiar (i.e., as homelike), since what was previously experienced as background now actively penetrates into our world, making our world appear as something to which we do not belong to. The balance between homelikeness and unhomelikeness is in such an episode of migraine temporarily distorted, such that our “not being at home in the world” becomes apparent, only to disappear from understanding when the migraine episode is no longer experienced.^1^ In other words, the experience of not being able to transcend *into* a meaningful world reveals the presence of a form of ordinary homelike attunement characterized by “the normal unapparent transparency of everyday activities” (Svenaeus 2013, p. 234).

Whether or not experiences of illness—such as migraine episodes—can be considered experiences of unhomelikeness depends on the extent to which they prevent returning into a mood of homelike attunement. When an episode of migraine has passed by, one often manages to return to an understanding of the world within which previous projects can be continued, and no major disruptions of experience has taken place. That is, one returns to the harmonious rhythm of healthy being-in-the-world (Ibid., p. 99). In contrast, experiences of illness—and this is what distinguishes them from episodes of temporary unhomelike attunement—disrupt the constitution of homelike attunement more permanently.

Phenomenological access to health is, on Svenaeus’s account, thus grounded in the ability to sense that one is no longer at home in the world that (s)he is attuned to, such that one is prevented from transcending into a meaningful world (2019, p. 464). Through this, the lack of self-evidence of the rhythm of one’s life can become an object of reflection, precisely because it is no longer a self-evident background attunement that one transparently lives through.

## Health and the interaction between subjective and objective body

Thus far, we saw that in contemporary phenomenology of medicine, there is a tendency to conceive of health as a way of being in the world in which our body remains transparent. At the same time, the possibility that our body ceases to be transparent (e.g., through the breakdown of bodily certainty or through episodes of unhomelike attunement) remains to exist. For Merleau-Ponty, it is precisely this characteristic of our bodily existence that is constitutive of how we experience the world: “What allows us to center our existence is also what prevents us from centering it completely, and the anonymity of our body is inseparably both freedom and servitude.[…] [T]he ambiguity of being in the world is expressed by the ambiguity of our body, and this latter is understood through the ambiguity of time” (Merleau-Ponty 2012, p. 87). The question addressed in this section is if we must necessarily conceive of this ambiguity as a burden (i.e., as a disruption of health).

The mere observation that our objective body bears the possibility of becoming transparent already indicates that our being in the world is constituted by a relationship between objective and subjective body that must not necessarily be conceived of as harmonious. The physiological processes taking place in the geometrical space of my objective body shape my experience. On top of that, it also influences my being in the world when I have knowledge of how certain processes in my body might influence my relationship with the world in the (near) future.

For example, a smoker can be confronted with a CT scan of her lungs and provided with knowledge that her behavior makes her lungs further deteriorate, resulting in an increased chance of having lung cancer. And as suggested, the number of times that we are confronted with this kind of knowledge is likely to increase in the near future due to the focus on personalized and preventive medicine. Alternatively, one can evaluate one’s own body without such a negative undertone. For example, one can be quite satisfied with one’s looks when seeing oneself in the mirror, or even be optimistic about the future after having obtained the results of a full body scan. Accordingly, observations of one’s own objective body help constituting judgments about whether or not one understands oneself as being healthy; expectations that might shape the habitual patterns that one searches to engage in. Such observations therefore not only allow one to be confronted with one’s physiology, but also temporalize one’s body. By projecting a specific past (e.g., by showing how smoking shapes the development of one’s lungs) and a specific future (by normatively determining the desirability of a specific habit in light of one’s physiology) onto one’s body, they point to how it is shaped by and might shape one’s being in the world.

Crucially, the observation of one’s own physiology does not take place by suddenly leaving one’s embodiment, such that a third-person perspective on a foreign object is established. Instead, “[t]he experience of one’s own body, however, reveals to us an ambiguous mode of existence. If I attempt to conceive of it as a bundle of third person processes […] I observe that these “functions” cannot be linked among themselves or to the external world through causal relations. All of them are confusedly taken up and implicated in a single drama. The body, then, is not an object” (Merleau-Ponty 2012, p. 204). When perceiving one’s own physiology, these perceptions are formed *from within* one’s own embodiment. They are embodied confrontations with something that is fundamental for the ongoing appearance of embodiment. Such confrontations show how one’s own objective body (and the way it endures) is shaped by the particular habits and projects one engages in. Accordingly, one’s body—despite allowing to be viewed *as* an objective body—is not an object itself, but is constituted precisely in the relationship between its being objective and its being subjective.

Yet, the fact *that* one’s objective body can be drawn out of its transparency—and is often drawn out of it, does not necessarily imply that such moments must be understood as part of being healthy. After all, it remains to be possible to interpret them as instances of breakdown (Carel 2016) or unhomelike attunement (Svenaeus 2001), such that it is only possible to experience the objective body when it starts becoming a burden we need to carry, instead of a medium *for* experience.

Wehrle makes a helpful distinction between two forms of embodied temporality that might make clear why experiencing one’s body as objective can be considered as a part of being healthy: “(i) the *intentional temporality* of *being* a body; and (ii) the *temporal experience* of *having* a body” (2019, p. 3). The former refers to the constitution of time *in* our relation with the world (e.g., by temporalizing the objects around us relative to ourselves and our goals and projects), whereas the latter points to the fact that our body as objective body is a temporal object already constituted *in* time (e.g., as temporalized by past events and perceptions). It is only because of the latter that it becomes possible to reflect explicitly on one’s action and habits, such that one’s lived body can be reflected upon.

The first form of embodied temporality constitutes my perception of the world around me in a certain way. Depending on my habits and skills, the objects around me appear as being easily or not easily to be integrated into my goals and projects. For example, the grasping of a smartphone as something that can be used to take a photograph of an old car (that might become part of my collection of photographs of old cars) requires me to have already grasped the rectangular object with a screen in my environment as contributing to this project. This grasp then is based on my previous interactions with smartphone-like looking objects such that I am habitually routinized of using them to take photographs. Accordingly, intentional temporality shapes how the world in terms of how I intend to operate on it relative to the goals and projects I intend to pursue. To put it in Merleau-Ponty’s terms, my body expresses an “I can”, thereby disclosing the world in such a way that I can unproblematically carry out the movements needed to pursue my goals and projects (2012, p. 139).

The second form of embodied temporality makes possible inferences about how the past of my objective body affects how intentional temporality is constituted, as well as projecting future forms of intentional temporality. Let me illustrate this with an example. When one attends the gym frequently, the particular parts of the body that one trains become increasingly muscular, such that the traces of embodied activity become a visible part of the objective body. Because of this, one might be surprised about the size and shape of one’s left upper arm, but can link this surprise to past embodied activities through which one’s arm eventually ended up being of a certain size and shape. Furthermore, one might experience that an increasingly large set of objects appear as carry-able, which one might recognize as resulting from exercising, making clear how changes in one’s own objective body are constitutive of a future embodied temporality.^2^

If the above analysis is correct, then there is no a priori reason to assume that situations in which the objective body is drawn out of its transparency cannot be incorporated into a phenomenological analysis of health. Instead, such experiences “mak[e] us aware of our bodies not only passively as material, changing and finite objects, but also actively, as possible bodies related to our future actions and plans” (Wehrle 2019, p. 20). As a result, it becomes visible that the objective body is fundamentally shaping our being in the world, and that changes in it might result in differences in how the world is disclosed to us.

## Technologies mediating the interaction between subjective and objective body

Let me move back now to how (preventive) medicine and the technologies it makes use of shape the experience of one’s own body. Technologies scanning our body form a crucial part of medical practice. For example, ultrasound is used to make images of the fetus in the womb of pregnant women, and magnetic resonance imaging or computer tomography are used to image specific organs. When being confronted with these scans, we are not confronted with images of an alien or external body, but instead with scans of our own body. As Svenaeus puts it, it is undeniable that such medical technologies “point towards the nature *in* me, or perhaps rather *of* me, that cannot be controlled” (2013, p. 236). In other words, such technologies do not offer neutral, objective images of just another object in the world, but instantiate a confrontation between something that is constitutive of one’s own individuality.

In postphenomenology, the term *technological mediation* is used to emphasize the non-neutrality of technologies that generate images (e.g., Ihde 1990; Rosenberger 2011).Technologies are understood as shaping how individuals experience and understand themselves and the world in which they are situated. They make present both the world and the individual relating to it in a specific way. From a phenomenological perspective it can therefore be said that technologies “codetermine how subjectivity and objectivity are constituted” (Verbeek 2005, p. 116). These technologies display what Ihde calls an *amplification-reduction* structure: they tend to amplify certain aspects of the phenomena being imaged, while turning away our attention from other aspects (Ihde 1979, p. 21). Accordingly, medical technologies mediate the relationship between subjective and objective aspects of the body, by making certain aspects of the body rather than others stand out.

Pointing to the technologically mediated way through which the objective body becomes present is important, because imaging technologies constitute it significantly different from moments in which I would touch it or see it in the mirror. While in the latter case, it become clear rather easily that I am confronted with my own body, because I can link the sensations of touch I experience to the touching body parts seen in the mirror, and can see my own movements when looking in a mirror. This kind of immediacy disappears in the case of the constitution of the objective body through imaging and modeling technologies used in medicine. Instead, I am being confronted with a presentation of an aspect of my objective body of which the meaning is not immediately transparent to me. Because of this, it becomes present as a site of hermeneutic inquiry.^3^

Such technological mediations previously were largely reserved to hospital settings. However, with the increased call for medicine to become a preventive medicine, citizens are urged by healthcare professionals to use other technological devices that establish a mediated relationship with their own bodies to become better aware of their own health status. For example, self-tracking applications allowing to monitor the daily number of steps one takes or one’s calorie intake are promoted as tools for disease prevention, because they allegedly help citizens to adopt a more healthy lifestyle. Also, the promise to develop so-called digital twins in the future, personalized models of myself and my biology that are being updated in light of measurements of my physiology and behavior, might give rise to an increase in the number of times in which I am being confronted with my own body through a particular technology. When using such technologies, a mediated relationship with my own body comes into being, such that the number of times in which my own body becomes a site of hermeneutic inquiry is likely to be greatly increased.^4^ Let me analyze these examples (i.e., self-tracking and the digital twin) in some more detail to show how different technologies mediate the relationship between subjective and objective body differently.

### Digital self-tracking

The practice of self-tracking in itself is not new. The monitoring and measuring of elements of one’s body or life for self-improvement is a practice having existed for a long time. One only has to think of how the diaries of Marcus Aurelius written in the second century AD helped him to keep track of his own life and critically evaluate his life choices and bodily feelings.

A more recent historical example of a technology explicitly mediating the relationship with the *body* by making aspects of it quantifiable is the weight scale. Originally being used as an attraction at carnivals were people weighed themselves publicly, this technology slowly moved into the intimacy of the houses of citizens in the beginning of the twentieth century, thereby losing its initially playful character (Crawford et al. 2015). Crucially—and this is what distinguishes the weight scale from keeping a diary, the weight scale was advertised as offering objective knowledge of one’s weight in contrast with subjective, experiential knowledge of one’s body. Especially in combination with tables identifying what would be ‘normal’ standards of height and weight, the weight scale became used to reach a specific ideal weight, thereby contributing to the normalization of certain body types over others (Ibid., p. 483). As a consequence, the weight scale mediates the relationship between subjective and objective body by making the latter explicitly present as an object that, when appreciated numerically, can function as an indicator of the (ab)normalcy of one’s own body.

The use and promotion of digital self-tracking (e.g., by using health monitoring applications) must be understood as a technological mediation giving rise to a different constitution of one’s own body than the weight scale did. On the one hand, this mediation takes place because of *what* is being measured by digital self-tracking technologies, namely some of the daily patterns in which users engage. They quantify one’s own body in a specific way by attempting to measuring how it is being *lived*, thereby promising not only to measure aspects of the objective body, but of one’s own body generally. When using them, “[w]e perceive in an embodied manner an however objectified version of our own embodiment” (Van Den Eede 2015, p. 151). On the other hand, one’s own body is constituted in a specific way through digital self-tracking because of *how* the collected data is already endowed with meaning by self-tracking applications. On the basis of the collected data, users receive feedback from self-tracking applications whether or not their behavior conforms to some pre-defined ideal standards.

Now, let me unpack the consequence of these two changes for the experience of our body in somewhat more detail. Two paradigmatic examples of digital self-tracking are measurements of the number of steps one takes on a daily basis and the monitoring of one’s calorie intake. *What* aspect of the body is amplified by such tracking devices? Most often, we do not explicitly count the number of steps we take, nor do we explicitly check the number of calories in our diets. Rather, we move from home to the supermarket, go for an afternoon stroll to clear the mind, or move from one meeting to another without paying explicit attention to the distances being covered. Such movements are habitual patterns integrated within the projects we undertake (e.g., going to a meeting) that we realize unproblematically. Similarly, the number of calories that we consume is constitutive of the state of our body, but often recedes from view when being engaged in the activity of eating or cooking. Yet, since we often have particular meals on a weekly basis, we habitually consume a certain amount of calories without us noticing. Hence, what is being measured and perceived by users of self-tracking applications is an aspect of their habitual way of being in the world executed *by* their own body having an effect *on* it. Put differently, self-tracking devices make the habits that are transparently lived *through* perceivable as objects that can be *related to*.

Zooming in on the feedback loops often integrated into self-tracking applications allows to see more in detail *how* the way data is used to offer feedback on certain habitual patterns constitutes one’s own body in a specific way. The data collected when using self-tracking applications not only is a way to passively observe certain habitual patterns, but also functions as a source for users that stimulates to undertake certain actions to change existing habits. To this purpose, feedback mechanisms are integrated into self-tracking applications that provide users with information with how well their habits conform to a certain standard. This standard can either be a pre-set general rule (e.g., 10.000 daily steps), or a goal one sets herself (I want to consume a certain amount of daily calories in order to gain or lose weight).

Applications can send motivational messages to users when they are in ‘danger’ not to realize a certain goal. For example, when working, I might receive a notification of my step-counter application that it is time for a walk when I want to fulfill the goal of taking 10.000 steps. Or, my calorie intake monitoring application might send me a message that I should refrain from consuming certain products if I want to lose or gain weight. Accordingly, my habitual patterns are not only turned into objects that I can relate to whenever it pleases me. Instead, I am being confronted with an objectified version of my habits while potentially being engaged in another project, and my habits are also interpreted for me by the applications used as (not) helping to realize a certain goal. In doing so, the applications attempt—on the basis of an objectified version of some of my habitual patterns—to motivate me to change my behavior on a consistent basis.

These two ways in which self-tracking applications mediate the constitution of the subjectivity and objectivity of the body. Self-tracking applications turn specific aspects of the body that previously were habits part of our subjective experience *of* the world into objective characteristics of our body that can be related to *in* the world. As a result, the temporal experience of having a specific body changes because my habits are turned into explicit objects of attention, such that I am confronted with how they might influence my future way of being in the world. In combination with the feedback that the applications offer, my body is presented as a possible project amongst others warranting care and attention. As such, it becomes present as both source and outcome of the habits we engage in and the supposed healthiness thereof.

However, because self-tracking applications often amplify specific health parameters (e.g., daily steps taken, calorie intake), the body is constituted as a particular kind of project. As a consequence, our attention is shifted away from several other aspects of one’s body that might be relevant for one’s health, such as the stress one experiences when attempting to bring one’s calorie intake back to a certain level.

### Digital twins

Besides potentially stimulating users to engage in a more healthy lifestyle, the data collected through self-tracking can also be used for more direct medical purposes. One of the ways in which researchers hope to use this data is for the development of so-called digital twins (or, digital patient avatars). Although this technology is still under development it is—given that large projects are currently being devoted to it (e.g., Health EU, Project Baseline)—worthwhile to consider how these digital twins might constitute a relationship with one’s own body.

Digital twins promise to offer “in silico representations of an individual that dynamically reflect molecular status, physiological status and lifestyle over time” (Bruynseels et al. 2018, p. 1). The successful development and implementation of such models is considered to be a crucial breakthrough for personalized and preventive medicine, because they bear the promise to make visually available in a 3D hologram—both to medical professionals and patients—the health status of an individual over time, and to observe how specific lifestyle choices and behavioral patterns affect one’s health status. The concept of “digital twin” originally emerged in the context of testing the functioning of jet engines by developing a dynamic model that reflects the architecture of a particular system and is able to predict the actual state of this system by monitoring its functioning. Such models, then, can be used for predictive maintenance: they are able to accurately stipulate when technical interventions might be useful—and the consequences of these interventions—way before anomalies will occur (Ibid., p. 3). Since these models are particularized for an individual system, they are able to detect and intervene when a system deviates from its ‘normal’ (‘healthy’) functioning. Now, based on the hypothesis that the human being can similarly be considered a complex—mechanical—system, it is sometimes stipulated that a similar kind of practice can be developed in personalized and preventive medicine (e.g., Liu et al. 2019; van Houten 2018: see also Health EU 2018).^5^

Currently, medical research is far from close to the development of an adequate in silico model of an individual human, let alone of the influence of specific lifestyle choices thereon. Yet, what is of interest here is how wearables for self-tracking are thought to be of use for mapping the development of one’s digital twin. It are precisely measurements such as the amount of daily steps one takes or of one’s heart rate that are needed to be able to develop a digital twin that develops dynamically over time—just as the human being that is modelled does (e.g., Gambhir et al. 2018). Let me now explore how the digital twin might mediate the constitution one’s own body by looking again how this mediation occurs due to (i) *what* is being measured, and (ii) *how* the collected data is endowed with meaning.

When one tracks the daily steps one takes and the calories one consumes, it becomes possible to visualize and gain insight into one’s daily behavioral patterns, such that the habits one executes become an objectified aspect of one’s own body that can be related to. This, then, is an objectified version of one’s own body that is *lived through*. However, in the case of the digital twin, such tracking activities can only appear as meaningful when being correlated with other measurements; in themselves they do not constitute the things to be integrated in the digital twin. For example, daily steps must be correlated with measurements of one’s heart rate, or calorie intake must be correlated with the precise working of one’s digestive system, such that they are in themselves no longer indicators of whether or not the human body is in a ‘healthy’ (i.e., ‘normal’) state. Furthermore, due to the complexity of the system being modeled, they cannot be identified as being (the most) relevant aspects for certain—positive or negative—changes in the system. Hence, what is being measured by self-tracking devices in this context are no longer patterns of habits one engages in, but rather potential indicators of a current or future unhealthy state. In this case, what is amplified is not the relevance of habitual patterns for one’s own body as being *lived*, but rather for the objective body as being detached from the concrete individual.

This change in *what* can be considered as a relevant measurement also structures *how* the collected data is endowed with meaning: one’s objective body is drawn out of its transparency in a very specific way, thereby constituted as a different site of hermeneutic inquiry. Whereas self-tracking applications can send motivational messages to users to change one’s behavior when one is in ‘danger’ not to fulfill a certain goal and leave it open to users whether or not to act upon those, the response to changes in one’s digital twin that threaten its normal functioning is not the motivation of an embodied individual, but instead an intervention into the (disembodied) objective body. The digital twin thus amplifies the body in terms of its objective aspects such that it primarily warrants a response in terms of the objectivity of biomedicine, thereby turning away attention from it as a site to which the subject can relate to.

It might be said that the above analyses contains a large “what-if” character, given that the digital twin is a technology far from being reality yet. However, by contrasting it with the current use of self-tracking technologies, it might become clear how technologies that amplify its objectivity mediate how one’s own body becomes hermeneutically accessible: they amplify objective aspects of one’s body as being disintegrated from experience, because the object is attributed the same temporality as other objects (such as a jet engine). In doing so, one’s body ceases to be one’s own, as the temporality in which it is evaluated is no longer connected to the *experience of* one’s own temporality.^6^ Through this technological mediation, one’s own body becomes present first and foremost as an object that that can be externally observed and intervened in.

## Conclusion: experiencing objectified health

At the beginning of this paper, it was suggested that the development of personalized and preventive medicine and the way it promotes self-measurements might constitute an inversion of the enigma of health (i.e., create a constant awareness of one’s health status). To what extent is this indeed the case in light of the analysis conducted in this paper?

One of the key features of self-tracking applications is that they specifically target habits that allegedly have a positive or negative effect on my health—and if the above analysis is correct, turn aspects of my health into objects that can be consciously attended to. I started this paper by offering two existing phenomenological accounts of health that describe health in terms of the harmonious alignment of objective and subjective body, such that our body becomes something that we transparently live *through.* Instead of an object that one can consciously attend to, health is, on these accounts, the implicit horizon *against which* our lives take place. Now, in what sense does the brief (post-)phenomenological analysis of how our experience of health is mediated by technologies challenge these assumptions?

First, by drawing on Wehrle’s distinction between the intentional temporality of being a body and the temporal experience of having a body, I showed that the objective and subjective aspects of one’s own body mutually constitute one another. Furthermore, I suggested that the intimate relationship between these two aspects not only becomes visible in cases of illness, but is part of the everyday experience of our body in health. Having a certain temporal experience of my body allows link these objective aspects of my body to my embodied intentionality. For example, when I have had a month without any boxing practice or other physical exercises, my temporal experience of my body changes because it starts to look and feel differently. Now, when after this month I continue boxing again, there might be a notable change in how I move through the boxing ring: my embodied intentionality is constituted differently. In such a case, the way my objective body is constitutive of my embodied intentionality becomes something that is experientially available to me, such that an aspect of my body ceases being something that I transparently live through.

Second, I showed how the relationship with one’s own body is mediated by the technologies used. Drawing from postphenomenology, I argued that what can be considered subjective and objective aspects of one’s own body are co-constituted by self-tracking devices, and are constituted differently in the case of digital twins. In the case of self-tracking applications that offer feedback on one’s executed habits, one’s own body becomes increasingly present as in need of attention of and care, thereby transforming it into a project amongst other possible projects. However, amplifying certain aspects of one’s health by objectifying them might render several other relevant aspects as subjective, and therefore outside of the scope of how one should work on one’s own health. This amplification/reduction structure of technologically mediated perception becomes even more clearly visible in the case of the digital twin (Ihde 1979, p. 21). Because the objective aspects of the body are amplified, it becomes increasingly difficult to conceive of it as a project that one can work on as a living subject. Instead, perceptions of the body are reduced to perceptions of the *objective body*, such that it is threatened to cease being *one’s own* body.

Third, both digital twins and digital self-tracking dramatize the split ambiguity of the body that is inevitably there. Through the use of self-tracking applications, our own body becomes present as a hermeneutic site that we are invited to evaluate in terms of certain habitual patterns that are correlated to health parameters. For example, we can start to appreciate the correlation between the current number of steps we take on a daily basis and the influence of this habitual pattern on the future constitution of our embodied intentionality. Accordingly, *if* our own body and the healthiness thereof are constituted through often implicit habitual patterns, and several of these patterns do have a bearing on our health status, then digital self-tracking constitutes a disharmony between objective and subjective body such that our own health becomes experientially accessible. To put it in Svenaeus’ terms, self-tracking invites explicit reflection on our current attunement with the world by making what was previously experienced as the background of our life as something actively penetrating into our life. On the contrary, the digital twin amplifies the split between objective and subjective body by interpreting health as something that belongs to one’s physiological system and that can be measured against the background of a ‘normal functioning’. In this sense, it does not invite users to reflect *on* how they are attuned with the world, but rather constitutes only the objective body as a site of hermeneutic inquiry.

By turning the objective body into a site of hermeneutic inquiry, objectified aspects of health cease from being an implicit horizon *through* which the world is disclosed, and are turned into explicit objects *in* the world. The experience of these aspects of one’s health *as* objectified is accompanied with the suggestion that particular habits might no longer be unproblematically executed, because of how they influence the future states that our body will be in. On the one hand, relating to one’s own body through self-tracking applications can be considered as a way to show how “[h]abit expresses the power we have of dilating our being in the world, or of altering our existence through incorporating new instruments” (Merleau-Ponty 2012, p. 145). The technologies used to disclose our habits can thus be considered as potentially constituting a new horizon against which our existence takes place. In this way, experiences of objectified health are fed back into one’s embodied existence such that they become part of the body one lives through. On the other hand, however, medical technologie—as demonstrated through the discussion of digital twins—might constitute a particular kind of horizon in which only the influence of our habits on our objective body is amplified. In this way, a horizon is constituted in which only particular things in the world stand out as meaningful while others do not. As a consequence, the possibility of relating to those is ruled out, such that also the possibility of reflecting on *why* one should develop habits that help fulfilling certain health parameters rather than others might disappear.

## Footnotes

1 This is not necessarily to say that a strict distinction between health and illness can be made in the absolute. Whether or not something can be qualified as illness is, according to Svenaeus, dependent on the extent to which such an experience gives rise to a long-term unhomelike attunement that prevents a person to project oneself into the future.

2 This is of course not to say that this new form of embodied temporality must necessarily be considered progress. The only point being made here is that changes in one’s objective body set new norms, such that an embodied “I can” might appear (or disappear) in particular situations.

3 In this paper, I focus on how *one’s own* objective body becomes a site of hermeneutic inquiry, instead of on how *an* objective body can become one for a medical professional that relates to it from a third-person perspective.

4 There is a vast amount of critical literature on self-tracking focusing and how it might threaten the autonomy of individuals. Criticism often focuses on how self-tracking applications have certain standards of normality inscribed in them, thereby enforcing a specific normative ideal (e.g., Devisch and Vanheule 2015; Lupton 2013), or on the amounts of highly individualized data that self-tracking applications collect that can be used by third parties for purposes of which users are unware, and which might conflict with the interests of users (e.g., Lanzing 2016). An extensive review of this literature is beyond the scope of this paper (but see Ruckenstein and Schüll [2017]), as the current focus is not on self-tracking proper, but instead on showing that health is constituted in the phenomenological relationship between subjective and objective body that is nowadays increasingly mediated by technologies.

5 In this paper, I am not going to develop an explicit argument to conceive of the human being and its health status as a mechanical model. I take it that the phenomenological approach developed in this paper sufficiently demonstrates the limits of a mechanical approach to the human body.

6 This is not to say that digital twin will necessarily *prevent* a patient from reflecting on how her habits might shape her future way of being in the world (i.e., establish a relation between subjective and objective body), but rather that they amplify the objectivity of one’s body.
